# Effect of high-flow nasal oxygen therapy combined with ipratropium bromide on Th1/Th2 balance and inflammation in patients with chronic obstructive pulmonary disease and respiratory failure

**DOI:** 10.12669/pjms.40.9.10221

**Published:** 2024-10

**Authors:** Ting Li, Xuefen Xu

**Affiliations:** 1 Ting Li, Department of Geriatric Medicine, Affiliated Nanjing Brain Hospital of Nanjing Medical University, Nanjing, Jiangsu Province 210029, P.R. China; 2 Xuefen Xu, Department of Geriatric Medicine, Affiliated Nanjing Brain Hospital of Nanjing Medical University, Nanjing, Jiangsu Province 210029, P.R. China

**Keywords:** High-flow nasal oxygen therapy, Ipratropium bromide, Chronic obstructive pulmonary disease, Respiratory failure, Th1/Th2, Inflammation

## Abstract

**Objective:**

To investigate the effect of a combination of high-flow nasal oxygen therapy (HFNO) and ipratropium bromide (IB) on Th1/Th2 balance and inflammation in patients with chronic obstructive pulmonary disease (COPD) and respiratory failure (RF).

**Methods:**

A retrospective analysis was conducted on the clinical data of patients with COPD and RF admitted to the Affiliated Nanjing Brain Hospital of Nanjing Medical University from June 2021 to March 2023. A total of 162 patients were included, with 79 patients received respiratory support using HFNO (HFNO group) and 83 patients who were treated using combined HFNO/IB (combined group). Treatment effect, lung function, levels of Th1/Th2, and inflammatory state were compared before and after the treatment.

**Results:**

Total effeicacy of patients in the combined group was significantly higher than that of the HFNO group (*P*<0.05). After the treatment, pulmonary function levels of the two groups was higher than that before the treatment, and was significantly better in the combined group compared to the HFNO group (*P*<0.05). The treatment was associated with a significant increase in the levels of Th1/Th2 in both groups. Post-treatment levels of these indexes in the combined group were markedly higher compared to the HFNO group (*P*<0.05). After the treatment, the inflammatory response of the two groups decreased, and was lower in the combined group that in the HFNO group (*P*<0.05).

**Conclusions:**

In COPD patients with RF, HFNO combined with IB is efficient in alleviating the inflammatory state of patients, restoring Th1/Th2 balance, and improving lung function compared to HFNO alone.

## INTRODUCTION

Chronic obstructive pulmonary disease (COPD) is the third leading cause of death worldwide, causing 3.23 million deaths in 2019.^1^ It mainly occurs in middle-aged and elderly people, and the incidence of patients over 40 years old is as high as 9.9% with a mortality rate of 9.7%.^1^-^3^ COPD patients often present with varying degrees of wheezing, chronic cough, and dyspnea.^3^,^4^ COPD exacerbations may lead to complications such as spontaneous pneumothorax and respiratory failure (RF), and RF is considered the most common adverse effect of COPD and one of the main causes of death.^3^-^6^43 in each group. Patients in the control group were orally administrated 20 mg of simvastatin, once a day. Patients in the observation group took 0.25g of azithromycin enteric-coated tablets, once a day, besides simvastatin. The treatment course of both groups was six months. Blood gas analysis indexes, forced expiratory volume in first second (FEV1 Studies show that impaired immunologic tolerance play an important role in pathogenesis of COPD,^7^,^8^ and COPD exacerbation and RF are associated with the imbalance of circulating CD4^+^ T cell subsets, more specifically, T-helper 1 and 2 (Th1/Th2) ratio.^1^-^4^ The balance of Th1/Th2 cells plays an important role in allergy and autoimmune diseases.

At present, clinical interventions for COPD patients with RF mainly include bronchiectasis, asthma relief, use of spasmolytic agents, and anti-infection therapy, but the overall effect is not satisfactory.^6^,^9^partial pressure of carbon dioxide (PaCO2 High-flow nasal oxygen therapy (HFNO), a noninvasive respiratory support that supplies heated and humidified gas mixture at a high flow through a nasal cannula, is commonly used in patients with COPD that is accompanied by RF.^10^ This method of respiratory support is simple and is able to supply high concentration of oxygen and efficiently alleviate the hypoxia.^9^,^10^ Ipratropium bromide (IB), anticholinergic drug, was shown to efficiently eliminate airway spasm in COPD patients with RF.^11^,^12^ However, the effect of HFNO combined with IB on the immune function, inflammation, and the Th1/Th2 balance in COPD patients with RF has not been widely confirmed. This study aimed to investigate the effect of HFNO combined with IB on the Th1/Th2 balance and inflammation in this population of COPD patients.

## METHODS

A retrospective analysis was conducted on the clinical data of patients with COPD and RF admitted to the Affiliated Nanjing Brain Hospital of Nanjing Medical University from June 2021 to March 2024. A total of 162 patients were included, with 79 patients who received respiratory support through HFNO (HFNO group) and 83 patients who received HFNO combined with IB (combined group).

### Ethical Approval:

The ethics committee of our hospital approved this study with the number NNL2024156, March 23^rd^, 2024.

### Inclusion criteria:

- Patients diagnosed with COPD and RF.^13^,^14^
- Patients with clear consciousness and stable vital signs.
- Patients with hemodynamic stability.
- The clinical data of the patients are complete.

### Exclusion criteria:

- Patients with nasal and facial injuries.
- Patients with pulmonary tuberculosis, chronic bronchitis and bronchial asthma.
- Patients with drug allergies.
- Patients with immune system diseases.
- Patients with mental disorders.
- Patients with malignant tumors.

### Respiratory support and treatment:

On admission, all patients were given symptomatic treatment such as bronchiectasis medications, nutritional support, anti-asthma and spasmolytic medications, anti-infection treatment, expectorant and cough relief agents according to specific conditions of each patient. (1) Patients in the HFNO group were treated with HFNO. The procedure was as follows which was performed using high-flow humidification oxygen therapy system (Airvo II, Fisher & Paykel, New Zealand). The relative humidity of the gas was set to 100%, the flow rate was 30 L/minute, the temperature was 37°C, and the concentration of inhaled oxygen was set to 40%. Oxygen concentration, temperature and flow were adjusted according to the specific conditions of the patients, and the duration of ventilation was ≥ 6 hours/day. Based on the HFNO, patients in the combined group also treated with IB. IB was administered as an aerosol inhalation of 1.25 ml IB (Boehringer Ingelheim Limited; Specification: 2.5 ml/piece) + 2 ml normal saline/time, three times/day. Patients in both groups were treated for a week.

### The following clinical indicators were collected:

- ***Baseline data:*** including gender, age, course of COPD, Apache-II score, RF classification, hypertension, hyperlipidemia, and smoking.
- Lung function index levels: the first second forced expiratory volume (FEV1), forced vital capacity (FVC), and FEV1/FVC were measured by the American Macquarie body mapping pulmonary function instrument (Model: ELITE DL).
- Serum Th1 and Th2 levels were measured by flow cytometry, and the Th1/Th2 ration was calculated.
- Inflammatory state was assessed by measuring levels of high-sensitivity C-reactive protein (hs CRP) and tumor necrosis factor -α (TNF-α) before and after the treatment using enzyme-linked immunosorbent assay.

### Treatment effect was classified into the following categories:

- Significant effect- expectoration and cough basically disappeared, blood gas status returned to normal, auscultation without wheezing sound, and normal breathing;
- Effective- expectoration and cough relieved, blood gas status significantly improved, wheezing disappeared or significantly reduced, mild dyspnea;
- Invalid-failure to meet the above standards. Total effective rate=significant effect rate+ effective rate.

### Statistical analysis:

Data were analyzed using SPSS version 26.0 (IBM Corp, Armonk, NY, USA). For continuous variables, mean and standard deviation (SD) were calculated. The independent sample *t*-test was used to compare the average value of two independent samples, and the paired t-test was used to determine the difference within the group over time. For categorical variables, the frequency distribution was provided and expressed as a percentage. The Chi square test was used to compare the classification variables between the two groups, such as treatment effect, gender distribution, hypertension, hyperlipidemia, and the history of smoking. A *p*-value less than 0.05 was considered statistically significant.

## RESULTS

**Fig.1:**
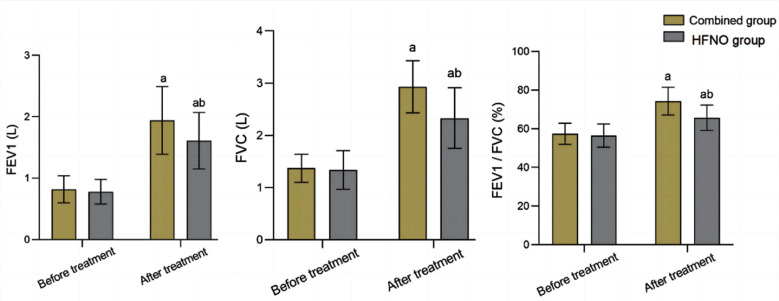
Comparison of lung function indexes between the two groups; IB: Ipratropium bromide; FEV1: forced expiratory volume in the first second; FVC: forced vital capacity; Compared with before treatment in the same group, ^a^P<0.05; Compared with IB group, ^b^P<0.05.

A total of of 162 COPD patients with RF (93 males and 69 females) met the inclusion criteria. Average age of the cohort was 68.02 ± 7.80 years (range, 52-85 years). There was no significant difference in baseline data between the two groups (*P*>0.05) (Table-I). The total effective rate of the combined group (96.39%) was significantly higher than that of the HFNO group (87.34%) (*P*<0.05) (Table-II). Before the treatment, there was no significant difference in FEV1, FVC and FEV1/FVC between the two groups (*P*>0.05). After the treatment, FEV1, FVC and FEV1/FVC in the two groups were higher than those before treatment, and markedly higher in the combined group compared to the HFNO group (*P*<0.05), Fig.1.

**Table-I:** Comparison of baseline data between the two groups.

Baseline data	Combined group (n=83)	HFNO group (n=79)	t/χ^2^	P
Male (yes)	46 (55.42)	47 (59.49)	0.275	0.600
Age (year)	68.72±7.51	70.24±6.79	1.349	0.179
Course of COPD (years)	6.49±2.38	6.15±2.56	0.876	0.382
APACHE II (score)	17.22±3.31	16.95±3.78	0.484	0.629
***RF classification***				
Type I	34 (40.96)	30 (37.97)	0.151	0.697
Type II	49 (59.04)	49 (62.03)		
Hypertension (yes)	35 (42.17)	32 (40.51)	0.046	0.830
Hyperlipidemia (yes)	11 (13.25)	17 (21.52)	1.934	0.164
Smoking (yes)	40 (48.19)	33 (41.77)	0.674	0.412

**Table-II:** Comparison of therapeutic effects between the two groups.

Group	n	Significant efficiency	Effective	Invalid	Total effective rate
Combined group	83	41 (49.40)	39 (46.99)	3 (3.61)	80 (96.39)
HFNO group	79	28 (35.44)	41 (51.90)	10 (12.66)	69 (87.34)
*χ^2^*					6.174
*P*					0.046

**Fig.2:**
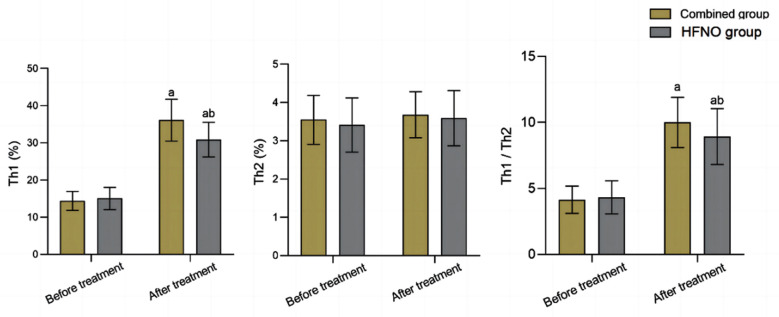
Comparison of Th1/Th2 levels between the two groups; IB: Ipratropium bromide; Compared with before treatment in the same group, ^a^P<0.05; Compared with IB group, ^b^P<0.05

**Fig.3:**
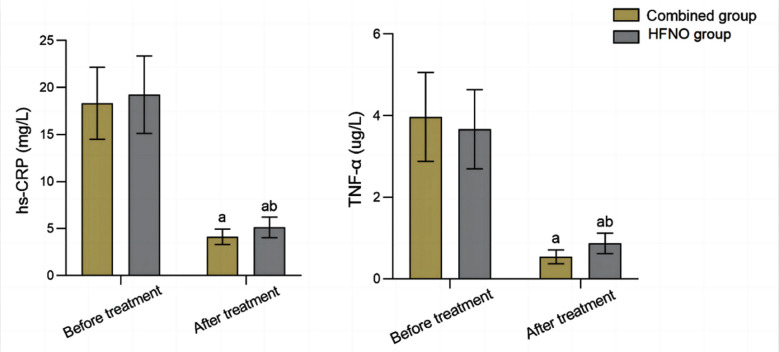
Comparison of microinflammatory state between the two groups; IB: Ipratropium bromide; hs-CRP: high-sensitivity C-reactive protein; TNF-α: Tumour necrosis factor alpha; Compared with before treatment in the same group, ^a^P<0.05; Compared with IB group, ^b^P<0.05.

Before the treatment, serum levels of Th1, Th2 and the Th1/Th2 ratio were comparable in the two groups (*P*>0.05). After the treatment, there was an increase in the levels of Th1 and Th1/Th2 in both groups, which were significantly higher in the combined group compared to the HFNO group (*P*<0.05), Fig.2. Pre-treatment levels of serum hs CRP and TNF-α were similar in the two groups (*P*>0.05). After the treatment, levels of serum hs CRP and TNF-α in both groups decreased, and were markedly lower in the combined group compared to the HFNO group (*P*<0.05), Fig.3.

## DISCUSSION

Both HFNO and IB are commonly used in clinical treatment of COPD with RF.^15^-^16^evidence to support widespread use of HFNO compared with non-invasive ventilation (NIV The study showed that a combination of HFNO and IB is significantly more effective in the treatment of COPD patients with RF compared to HFNO alone. Sun et al.^17^ evaluated the efficiency of noninvasive positive pressure ventilation and HFNO in treating patients with COPD and RF, and showed that the success rate of the two groups was 28.2%, and the 28 days mortality rate was 14.0% and 15.4%, respectively. However, the incidence of adverse events in patients treated with HFNO was 5.1%, significantly lower than that of noninvasive positive pressure ventilation (20.9%). Additionally, studies show that the comfort scores of patients with COPD and RF treated with HFNO are higher than those of patients on noninvasive positive pressure ventilation.^18^,^19^ The long-term follow-up study of the application value of HFNO in COPD patients with RF showed that HFNO can effectively reduce the frequency of acute exacerbation of COPD, lower the number of readmissions, and is associated with a satisfactory prognosis.^20^,^21^ Lin et al.^22^ also showed that a combination of HFNO and IB was associated with better improvement of lung function and blood gas status compared to patients treated with simple ventilation.

Moreover, levels of inflammatory factors and the incidence of adverse reactions in patients who received the combined treatment were lower than those of patients treated with a simple ventilation. Similarly, treatment effect and quality of life improvement effect was better compared to simple ventilation treatment. Our results further confirm these findings. We showed that total efficacy of the combined group after the treatment was significantly higher compared to HFNO alone. Combined treatment in our study was associated with superior improvement in lung function and levels of inflammatory factors compared to simple HFNO method. Jiang et al.^23^ also confirmed that combined HFNO/IB regimen was able to more effectively alleviate inflammatory state in COPD patients, and was associated with better treatment effect and more pronounced improvement in lung function compared to HFNO alone. We may speculate, therefore, that the observed improvement in the treatment efficiency may be due to the unique properties of IB. Ipratropium is a bronchodilator medication that dilates the airways of the lungs, it can be quickly absorbed by the body, effectively and stably promotes sputum discharge, regulates the tension of the vagus nerve, reduces the production of inflammatory transmitters and airway secretions, inhibits M-receptor of airway smooth muscle, relaxes smooth muscle, and reduces bronchospasm.^24^,^25^ It can improve the lung function of patients with COPD by nebulized inhalation^26^ and long been confirmed that extended therapy with ipratropium is associated with improved lung function in patients with COPD.^27^

In this study, post-treatment levels of Th1 and the Th1/Th2 ratio of the combined group were higher than those of the HFNO group. Th1 and Th2 interact with each other and act synergistically to maintain the balance of immune function.^28^,^29^ Sun et al.^30^ found that COPD is associated with imbalanced Th1/Th2 ratio: while the level of Th2 cells remained unchanged, the level of Th1 cells was markedly lower, leading to aggravated lung function injury. Our results further confirm that HFNO combined with IB has high application value in COPD with RF, and is conducive to correcting Th1/Th2 imbalance, restoring immune function, and ensuring good prognosis of the disease.

The main strength of this study is that it allows to bridge an existing evidence gap in understanding the effect of the combined HFNO/IB treatment on the Th1/Th2 balance before and after the treatment. Additionally, we provide compelling evidence of the superiority of HFNO combined with IB on the lung function, inflammatory response and therapeutic effect in COPD patients with RF.

### Limitations:

This is a single center retrospective study with a small sample size and selection bias. Additionally, there was no follow-up management for patients. Therefore, further studies are needed to determine whether the research results are representative and the confirm the impact of the combined treatment on the prognosis of COPD patients with RF.

## CONCLUSION

Compared with HFNO alone, HFNO combined with IB can more effectively alleviate inflammatory state, restore Th1/Th2 balance, improve lung function, and the overall intervention effect in COPD patients with RF.

### Authors’ contributions:

**TL:** Conceived and designed the study.

**TL** and **XX:** Collected the data and performed the analysis.

**TL:** Was involved in the writing of the manuscript and is responsible for the integrity of the study.

All authors have read and approved the final manuscript.
